# Association of cilioretinal artery perfusion length with central visual acuity and vascular circulation in central retinal artery occlusion

**DOI:** 10.1186/s40942-026-00921-3

**Published:** 2026-08-17

**Authors:** Ke Wu, Tao Lei, Runsheng Wang, Bo Zhang, Weiwei Wang, Xiaotang Wang, Teng Ma

**Affiliations:** Shaanxi Eye Hospital, Xi’an People’s Hospital (Xi’an Fourth Hospital), Affiliated People’s Hospital of Northwest University, Xi’an, 710004 China

**Keywords:** Central retinal artery occlusion(CRAO), Cilioretinal artery, Best-corrected visual acuity(BCVA), Fluorescein fundus angiography(FFA)

## Abstract

**Purpose:**

This study aims to investigate the effects of varying lengths of cilioretinal artery perfusion on central visual acuity and fundus angiography circulation time in patients with central retinal artery occlusion (CRAO).

**Methods:**

A retrospective analysis included 582 CRAO patients, including 87 patients with cilioretinal arteries. Cilioretinal arteries were classified by direction into nasal and temporal groups, with the temporal group further subdivided into four subtypes based on the optic disc-macula distance (DMD): 1/4, 2/4, 3/4, and 4/4 DMD. Differences in basic information, visual acuity, and circulation time among the groups were analyzed.

**Results:**

Patients in the 4/4 DMD group were the oldest among the temporal subgroups (*P* < 0.05). The 4/4 DMD group demonstrated the best BCVA, while the Nasal-1/4DMD group had the worst (*P* < 0.05); notably, 13.33% of patients in the 4/4 DMD group had no light perception. There was no statistically significant difference in the circulation time of four groups (both arm-retinal circulation time and retinal trunk-to-branch filling time, *P* > 0.05).

**Conclusion:**

CRAO patients with cilioretinal artery supply less than 1/4 DMD have poor outcomes. 4/4DMD group was associated with better visual outcomes, but extensive macular perfusion by the cilioretinal artery did not invariably prevent severe visual loss during the acute phase.

**Supplementary Information:**

The online version contains supplementary material available at https://doi.org/10.1186/s40942-026-00921-3.

## Introduction

Central retinal artery occlusion (CRAO) is an ophthalmic emergency caused by acute retinal ischemia due to occlusion of the central retinal artery, leading to rapid or permanent vision loss in the affected eye [1]. However, when a cilioretinal artery is present, it appears to be somewhat protective of vision [2, 3].

The cilioretinal artery originates from the short posterior ciliary arteries or the Zinn- Haller ring, and usually extends to the macula region [4, 5] The potential protective effect of cilioretinal artery presence on visual acuity outcomes after CRAO has been reported previously [6–8]. For example, Kim et al. examined 90 patients with CRAO and found that patients with cilioretinal arteries demonstrated significantly improved initial and final visual acuity outcomes compared to those without such arteries, though overall visual outcomes remained poor [6]. Additionally, there are several case reports of CRAO with preserved cilioretinal artery [7–10].

Previous studies have demonstrated that the presence of cilioretinal arteries plays a positive role in preserving visual acuity following CRAO onset, particularly those supplying the macular region of the retina [2, 3]. However, cilioretinal arteries show substantial individual variability in both direction and extent of macular reach. Whether cilioretinal arteries with different courses or degrees of extension toward the macula provide comparable protective effects remains unclear, and relevant data on this issue are currently limited.

Therefore, in this paper, we included a large number of CRAO patients and aimed to investigate the effects of varying lengths of cilioretinal artery perfusion on central visual acuity and fundus angiography circulation time.

## Methods

### Participants

This study retrospectively analyzed patients hospitalized at the Neuro-Ophthalmology Center of Xi’an People’s Hospital (Xi’an Fourth Hospital) from May 2019 to May 2024 who met the diagnosis of CRAO. The study was approved by the Ethics Review Committee of Xi’an People’s Hospital (Xi’an Fourth Hospital) (Approval No: 20220005). The requirement for informed consent was explicitly waived by the Ethics Committee, as this was a retrospective study involving only anonymized clinical and imaging data, and no additional patient contact or intervention was required.

We collected age, gender, time from CRAO onset to hospital, best-corrected visual acuity (BCVA) at admission, fundus color photography and fluorescein fundus angiography (FFA), and magnetic resonance images (MRI) or computed tomography (CT) of the head and orbits from all CRAO patients. (The imaging examination results are evaluated by radiologists, neurologists, and ophthalmologists.) Patients with bilateral CRAO were excluded, and only the affected eye from each patient with unilateral CRAO was included in the analysis.

The inclusion criteria for patient selection were as follows: (1)color fundus photography revealing grayishwhite edema in the posterior pole, (2)fluorescein fundus angiography (FFA, Spectralis HRA, Heidelberg, Germany) indicating a delayed arm retinal circulation time(ARCT) exceeding 15 s(s) and/or an extended time of more than 2s for reitinal trunk-to-branch filling time(FT), potentially accompanied by retrograde filling, faint fluorescein flow, segmental filling patterns, and with or without fluorescein leakage [11].

**Fig. 1:**
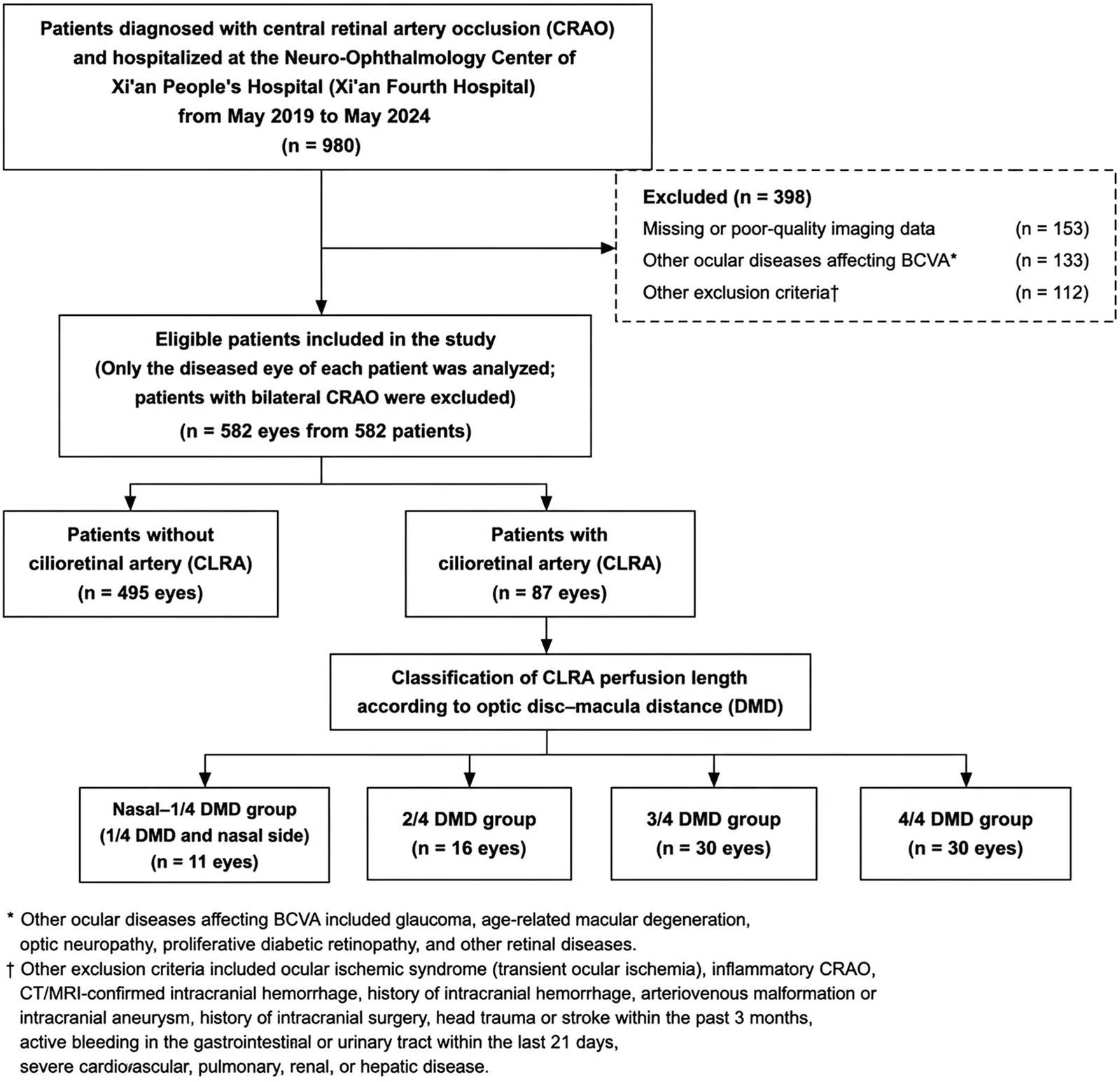
Flowchart of patient enrollment and study design

Exclusion criteria included: (1)Patients with missing imaging data or poor image quality; (2)Concurrently associated with other ophthalmic conditions affecting BCVA, such as glaucoma, age-related macular degeneration, optic neuropathy, proliferative diabetic retinopathy, and so forth; (3)ocular ischemic syndrome (transient ocular ischemia); (4)inflammatory CRAO; (5)CT/MRI-confirmed intracranial hemorrhage, (6)a history of intracranial hemorrhage, arteriovenous malformation, or intracranial aneurysm; (7)a history of intracranial surgery, head trauma, or stroke in the past 3 months, (8)active bleeding history in the gastrointestinal or urinary tract within the last 21 days, (9)severe cardiovascular, pulmonary, renal, or hepatic disease [12]. (The patient selection process is illustrated in Fig. 1.)

### Assessment criteria

FFA methods: Fluorescence angiography was performed by a Heidelberg fundus fluorescence angiograph according to the fundus fluorescence angiography protocol, and a timer was started at the same time as the fluorescein contrast agent was injected to record the prearterial arterial phase of the fundus retinal arteries in the early venous phase (venous double-tracking phenomenon) and the mid- and late venous phases up to the late phase of about 10 min. Uniformly trained technicians at our institution performed all imaging videos.

On FFA, the ciliary choroidal artery can be identified based on its filling sequence and morphological characteristics. Originating from the posterior ciliary artery, it appears 0.5–1.5 s(s) earlier than the central retinal artery (CRA) and typically manifests during the choroidal phase. Morphologically, these arteries originate from the optic disc margin and follow a relatively straight course. They do not exhibit the CRA’s characteristic centripetal radial branching pattern but instead supply blood to localized retinal regions [13, 14] Therefore, the presence, direction of travel, and length of the cilioretinal artery in this study were determined by FFA video recording. Previous studies have classified CRAO into incomplete, subtotal, and complete forms based on the overall severity of retinal ischemia using clinical findings and multimodal ophthalmic examinations [15]. Because no universally accepted quantitative criteria exist to clearly distinguish these categories, we combined incomplete and subtotal CRAO into a single incomplete occlusion group for the purpose of this study. Because no universally accepted quantitative criteria exist for distinguishing complete and incomplete CRAO, we adopted operational criteria based on previous work from our group [16]. Eyes with an ARCT ≥ 35 s and/or FT ≥ 35 s on FFA were classified as complete CRAO, whereas the remaining eyes were classified as incomplete CRAO. BCVA was assessed using the International Standard Visual Acuity Scale (ISVAS) and converted to the logarithm of the minimum angle of resolution (logMAR) format [17–19].

**Fig. 2:**
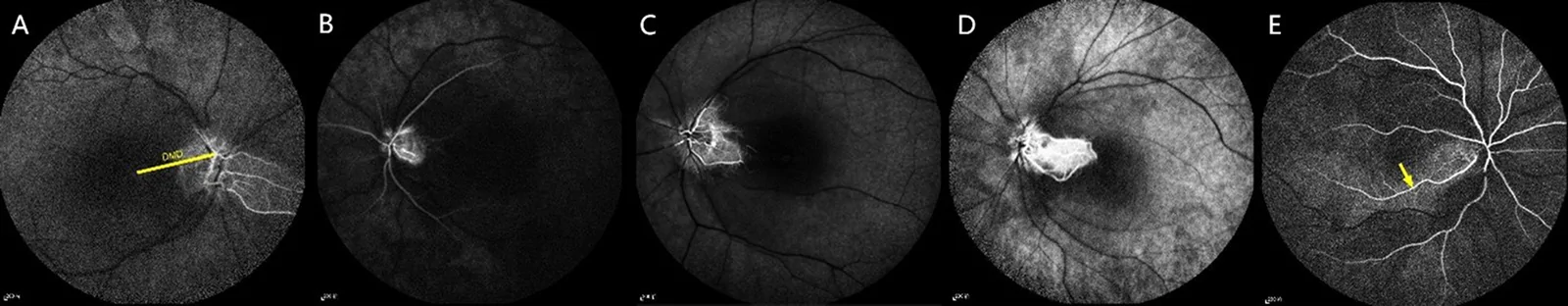
Five groups of cilioretinal artery types are shown. Figure **A** shows the nasal group, and figures **B**-**E** show the temporal (macular) group. The yellow line in Figure **A** indicates the optic disc macular distance (DMD). Figure **B** shows group 1/4DMD, Figure **C** shows group 2/4DMD, Figure **D** shows group 3/4DMD, and Figure **E** shows group 4/4DMD, with yellow arrows marking the cilioretinal artery

Criteria for dividing the scope of supply of cilioretinal arteries: In this study, cilioretinal arteries were classified according to their direction of course as either nasal (Fig. 2A) or temporal (macular; Fig. 2B and E). Temporal cilioretinal arteries supplying the macular region were further categorized into four subgroups based on the ratio of the arterial extension length to the optic disc–macula distance (DMD), defined as the distance between the center of the optic disc and the foveal center. They were group 1/4 (Fig. 2B): cilioretinal artery length was 1/4 or less than 1/4 of DMD, group 2/4 (Fig. 2C): cilioretinal artery length was longer than 1/4 DMD but not more than 2/4 DMD, group 3/4 (Fig. 2D): cilioretinal artery length is longer than 2/4 DMD but not more than 3/4 DMD, Group 4/4 (Fig. 2E): cilioretinal artery length is more than 3/4 DMD and even beyonds the macular foveal. Given that nasal cilioretinal arteries did not extend toward the foveal region, they were grouped together with temporal arteries reaching 1/4 DMD, namely Nasal-1/4DMD group. 

Two researchers (who were licensed ophthalmologists) independently classified the group of cilioretinal artery using FFA images. A senior expert in fundus diseases resolved disagreements.

### Statistical analysis

Data that conformed to normally distributed measures were expressed as mean± standard deviation, comparisons between two groups were conducted by independent samples t-test, comparisons of data between multiple groups were conducted by one-way analysis of variance(ANOVA), multiple comparisons that satisfied variance alignment were analyzed by least significant difference(LSD), and multiple comparisons that did not satisfy variance alignment were analyzed by Tamhane’s T2 test; data that did not conform to normal distribution was expressed as median (interquartile spacing) and analyzed using nonparametric tests. Count data were expressed as the number of cases (percentage), and comparisons between groups were made using the chi-square test. The consistency test used the Kappa coefficient test.

All statistics were analyzed using SPSS 29.0 (SPSS, Inc., Chicago, IL, USA). Propensity score matching (PSM) was performed using the MatchIt package for the R project [20] (R version 4.2.1, R Foundation for Statistical Computing, Vienna, Austria; http://www.r-project.org). *P* < 0.05 was considered statistically significant in this study.

## Results

### Characteristics of the patients

We initially identified 980 patients with CRAO. Of these, 153 patients (15.61%) were excluded because unclear FFA images or poor cooperation precluded reliable identification and classification of the cilioretinal artery. 133 patients (13.57%) were excluded because coexisting ocular diseases could independently affect BCVA and confound the assessment of visual outcomes. Another 112 patients (11.43%) were excluded based on other predefined criteria, including intracranial space-occupying lesions, cerebral hemorrhage, or other conditions that could interfere with ophthalmic evaluation. Ultimately, 582 patients (eyes) (59.39%) met the eligibility criteria and were included in the final analysis. Among the 582 patients, 87 patients (14.95%) with cilioretinal artery and 495 patients (85.05%) without cilioretinal artery (as shown in Fig. 1), mean age 57.80 ± 13.18years, 341(58.59%)males, 277(47.59%) right eyes, time from CRAO onset to hospital 63.21 ± 58.95 h, CRA incomplete occlusion 358 cases (61.51%). Of the 495 patients without a cilioretinal artery, the mean age was 57.92 ± 13.11 years, 310 (62.63%) had incomplete CRAO, and the time from CRAO onset to hospital was 59.89 ± 52.64 h. Of the 87 patients with cilioretinal artery, the mean age was 57.13 ± 13.63 years, 48 (55.17%) had CRA incomplete occlusion, and the time from CRAO onset to hospital was 82.09 ± 84.48 h.

Because of the significant difference in the number of patients and population characteristics between the two groups, we screened the two groups using PSM analysis 1:1 based on the severity of CRA occlusion, the time from CRAO onset to hospital, and age composition to further investigate the effect of the presence of the cilioretinal artery on visual acuity of patients with CRAO (Table 1).

From Table 1, we conclude that patients with cilioretinal arteries had better BCVA than those without cilioretinal arteries (*P* < 0.05), with no differences in age, time from CRAO onset to hospital, or CRAO severity. Indicating that the presence of cilioretinal arteries plays a beneficial role in the occurrence of CRAO.

Two researchers independently classified the cilioretinal artery supply range in 87 patients (87 eyes) using FFA images. A senior expert in fundus diseases resolved disagreements. The results showed a high degree of consistency between the two researchers in classifying the cilioretinal artery’s supply areas. (*P* < 0.001, Kappa coefficients of 0.795, see Supplementary Table 1).

**Table 1:** Comparison of basic information after PSM in patients with and without cilioretinal arteries

	With cilioretinal artery	Without cilioretinal artery	*P*
Number	87	87	-
Severity of CRAO (complete occlusion%)	39(44.8%)	45(51.7%)	> 0.05
Time from CRAO onset to hospital(hours)	82.09 ± 84.48	74.32 ± 74.98	> 0.05
Age(years)	57.13 ± 13.63	56.87 ± 13.16	> 0.05
BCVA, Snellen equivalent (logMAR)	20/1800(1.96 ± 0.72)	20/2800(2.15 ± 0.69)	0.037

### Effect of different cilioretinal artery length on BCVA of CRAO

We included a total of 87 patients with cilioretinal artery, of which the Nasal-1/4 DMD group had 11 cases (12.64%, including 2 cases of the nasal side), the 2/4 DMD group had 16 cases (18.39%), and the 3/4 DMD group had 30 cases (34.48%). The 4/4 DMD group had 30 cases (34.48%). As shown in Table 2, there was a statistically significant difference in age between the groups (*P* < 0.05). The age of the patients in the 4/4DMD group was 62.53 ± 14.47 years, which was older than other groups, especially the 3/4 DMD group(*P* < 0.05). There was no statistically significant difference in the time from CRAO onset to hospital or in the severity of CRAO across the four groups (*P* > 0.05).

There was a statistical difference in BCVA (logMAR) between the four groups (*P* < 0.05; Table 2). The BCVA (logMAR) in the 4/4DMD group was 1.57 ± 0.96, which was superior to the remaining three groups (Nevertheless, four patients in the 4/4 DMD group had NLP visual acuity), and the BCVA(logMAR) in the Nasal-1/4 DMD group was 2.37 ± 0.59, which was the worst group(Table 2). However, when comparing between-group differences, there was no statistically significant difference in BCVA between the 3/4 DMD group and the 4/4 DMD group.(*P* = 0.072,using the Tamhane’s T2 test).

In addition to between-group comparisons, a Jonckheere–Terpstra trend test demonstrated a significant monotonic trend toward better BCVA with increasing cilioretinal artery perfusion length (standardized J-T statistic = − 4.427, *P* < 0.001).

**Table 2:** Comparison of basic information and BCVA in different cilioretinal artery lengths

	Nasal-1/4DMD	2/4DMD	3/4DMD	4/4DMD	*P*
Number	11	16	30	30	-
Age(years)	53.82 ± 10.10	58.44 ± 14.98	52.23 ± 11.40	62.53 ± 14.47	0.022
Severity of CRAO (complete occlusion%)	6(54.5%)	7(43.8%)	12(40.0%)	14(46.7%)	> 0.05
Time from CRAO onset to hospital(hours)	66.55 ± 49.00	59.44 ± 62.76	92.27 ± 113.04	89.60 ± 70.66	> 0.05
BCVA, Snellen equivalent (logMAR)	20/4700(2.37 ± 0.59)	20/3300(2.22 ± 0.37)	20/2400(2.07 ± 0.40)	20/800(1.57 ± 0.96)	0.001

### Effect of different cilioretinal artery length on retinal vascular circulation of CRAO

We analyzed FFA imaging videos from 87 patients, which were adjudicated by a senior fundopathologist for individual circulation times (Table 3), including the time of cilioretinal artery filling, ARCT, and FT. By statistical analysis, we found that there was no statistically significant difference in all vascular circulation times in the four groups(Table 3, *P* > 0.05).

**Table 3:** Differences in retinal vascular circulation in five groups

	Nasal-1/4DMD	2/4DMD	3/4DMD	4/4DMD	*P*
Cilioretinal artery filling time(s)	14.36 ± 3.91	15.56 ± 5.51	15.80 ± 3.70	16.17 ± 4.32	> 0.05
ARCT(s)	38.36 ± 15.15	29.38 ± 12.56	29.13 ± 8.50	30.77 ± 13.21	> 0.05
FT(s)	28.73 ± 20.16	18.75 ± 17.45	21.20 ± 18.50	20.73 ± 19.16	> 0.05

## Discussion

Both the cilioretinal artery and the CRA branch off from the ophthalmic artery and are terminal vessels that do not anastomose with each other [21]. The cilioretinal artery is an anatomic variant of the arterial vasculature that emanates either directly from the short posterior ciliary artery or from the arterial circle of Zinn-Haller, which is part of the posterior ciliary artery feeder, and distributes through the margins of the optic disc to the macula and the retina beyond it. The prevalence of cilioretinal arteries in the Han population of northern China is estimated to be around 35%, with the majority supplying the temporal retina (78.3%), and a smaller proportion contributing to the nasal retinal circulation (14.8%) [22]. Hayreh et al. noted that the prevalence of CRAO with preserved cilioretinal arteries ranged from 14.0% to 26.0% [23]. This study found that patients with cilioretinal arteries had better visual acuity at the onset of CRAO than those without cilioretinal arteries, suggesting that the presence of cilioretinal arteries may contribute to the preservation of central visual function after CRAO [24]. Previous studies have also suggested a protective role of cilioretinal artery perfusion in maintaining central visual function in other ocular conditions, such as open-angle glaucoma [25].

There were individual differences in the range of supply of the cilioretinal artery, with most (97.70%) reaching the temporal side of the optic disc in this study, and 2 cases had nasal cilioretinal arteries. The BCVA was better in the patient with the temporal cilioretinal artery than in the two patients with the nasal cilioretinal artery, so we believe that the cilioretinal artery on the nasal side is only protective for the retina it feeds and is not very significant for the preservation of central vision. In addition, we divided the temporal cilioretinal arteries into four subgroups based on the relationship between the extent of cilioretinal artery supply and the macular fovea. We showed that patients with cilioretinal arteries covering the macular fovea (group 4/4DMD) had the best visual acuity, especially better than those in group 2/4DMD, in addition to the between-group comparisons, trend analysis demonstrated a significant monotonic association between increasing cilioretinal artery perfusion length and improved BCVA, further supporting the prognostic value of this anatomical classification. Notably, although patients in the 4/4 DMD group were older on average, they paradoxically demonstrated better visual outcomes after disease onset, which contrasts with the conventional understanding that advanced age is generally associated with poorer prognosis. We speculate that this inverse trend may be attributable to the larger perfusion territory provided by the cilioretinal artery in the 4/4 DMD group. Following the onset of CRAO, a more extensive cilioretinal arterial supply may maximally reduce ischemia and hypoxia of the inner retinal layers, thereby better preserving retinal ganglion cell function and resulting in improved visual outcomes. Consequently, even in older patients, sufficient cilioretinal artery perfusion may play a critical protective role in post-onset visual acuity.

Our study analyzed fundus vascular circulation in FFA imaging videos. No statistically significant difference in cilioretinal artery filling time was found among the groups. We hypothesize that during CRAO, CRA pressure drops abruptly. At this point, blood pressure within the posterior ciliary artery system (including the cilioretinal artery) remains relatively unaffected, and these vessels continue to fill at a relatively normal perfusion pressure. There were no statistically significant differences in ARCT and FT times among the five groups, indicating that the perfusion area of the cilioretinal artery does not influence the vascular circulation of the retinal artery. This finding is consistent with our established understanding.

Interestingly, four patients (13.33%) in the 4/4 DMD group presented with NLP despite having a cilioretinal artery supplying the entire macular region. We speculate that this apparently paradoxical finding may be explained by several factors. Although the cilioretinal artery anatomically perfused the macula, its perfusion pressure and blood flow may have been insufficient to fully compensate for the abrupt interruption of retinal circulation caused by complete CRAO. Consequently, anatomical preservation of macular perfusion may not provide adequate physiological support during the acute ischemic phase [6, 26]. In addition, severe acute retinal ischemia may induce profound but potentially reversible retinal neuronal dysfunction before irreversible structural damage occurs. Notably, all four patients experienced visual improvement after intra-arterial thrombolysis, supporting the possibility that the initial NLP reflected severe ischemia-induced functional suppression rather than irreversible retinal injury. Therefore, the protective effect of a long cilioretinal artery may not necessarily preserve visual function immediately after the onset of complete CRAO, but instead may enhance retinal survival and increase the potential for visual recovery following reperfusion.

Of course, this study has some limitations. Firstly, this was a single-center retrospective study, which may have introduced selection bias and limited the generalizability of our findings. In addition, approximately 40% of the initially identified CRAO cases were excluded based on predefined eligibility criteria, Although these exclusions were necessary to ensure accurate classification of cilioretinal artery perfusion and reliable assessment of visual outcomes, they may have further introduced selection bias and reduced the applicability of our findings to patients with poor imaging quality or significant ocular comorbidities. Therefore, the results should be interpreted with caution when extrapolated to broader CRAO populations. Future prospective multicenter studies with broader inclusion criteria are warranted to validate our findings. Nextly, the classification of CRAO severity based on FFA parameters remains imperfect. Since retinal perfusion impairment occurs along a continuous spectrum, the thresholds used to define complete and incomplete CRAO may not represent absolute pathophysiological boundaries. Therefore, these criteria should be interpreted as practical operational definitions rather than definitive biological classifications. And lastly, we included cycle times derived from FFA video recordings, and although all the technicians underwent a uniform training, we cannot exclude the possibility of incidental errors caused by inter-individual operating habits and the severity of the patient’s cooperation.

## Supplementary Information

Below is the link to the electronic supplementary material.

Supplementary Material 1

## Data Availability

No datasets were generated or analysed during the current study.
