# Supplementary material for: Association of cilioretinal artery perfusion length with central visual acuity and vascular circulation in central retinal artery occlusion
Source: Int J Retina Vitreous. 2026 Aug 17;12:132. doi: 10.1186/s40942-026-00921-3 (PMC13621633; doi:10.1186/s40942-026-00921-3)
Supplement: Supplementary file 1 [file 40942_2026_921_MOESM1_ESM.docx]

**Supplemental content:**

Supplementary Table 1: Consistency Tests Between researchers A and B for classifying the supply areas of the cilioretinal artery

|  |  | Researcher B | | | | |
| --- | --- | --- | --- | --- | --- | --- |
|  |  | 1/4DMD | 2/4DMD | 3/4DMD | 4/4DMD | Nasal |
| Researcher A | 1/4DMD | 8 | 2 | 0 | 0 | 0 |
|  | 2/4DMD | 2 | 13 | 2 | 0 | 0 |
|  | 3/4DMD | 0 | 3 | 25 | 3 | 0 |
|  | 4/4DMD | 0 | 0 | 1 | 26 | 0 |
|  | Nasal | 0 | 0 | 0 | 0 | 2 |
